# Systematical Investigation on Anti-Fatigue Function and Underlying Mechanism of High Fischer Ratio Oligopeptides from Antarctic Krill on Exercise-Induced Fatigue in Mice

**DOI:** 10.3390/md22070322

**Published:** 2024-07-19

**Authors:** Sha-Yi Mao, Shi-Kun Suo, Yu-Mei Wang, Chang-Feng Chi, Bin Wang

**Affiliations:** 1 Zhejiang Provincial Engineering Technology Research Center of Marine Biomedical Products, School of Food and Pharmacy, Zhejiang Ocean University, Zhoushan 316022, China; msy1361209282@163.com (S.-Y.M.); 13275896859@163.com (S.-K.S.); wangyumei731@163.com (Y.-M.W.); 2 National and Provincial Joint Engineering Research Centre for Marine Germplasm Resources Exploration and Utilization, School of Marine Science and Technology, Zhejiang Ocean University, Zhoushan 316022, China

**Keywords:** high Fischer ratio oligopeptides (HFO), Antarctic krill (*Euphausia superba*), anti-fatigue, in vivo metabolites, oxidative stress

## Abstract

High Fischer ratio oligopeptides (HFOs) have a variety of biological activities, but their mechanisms of action for anti-fatigue are less systematically studied at present. This study aimed to systematically evaluate the anti-fatigue efficacy of HFOs from Antarctic krill (HFOs-AK) and explore its mechanism of action through establishing the fatigue model of endurance swimming in mice. Therefore, according to the comparison with the endurance swimming model group, HFOs-AK were able to dose-dependently prolong the endurance swimming time, reduce the levels of the metabolites (lactic acid, blood urea nitrogen, and blood ammonia), increase the content of blood glucose, muscle glycogen, and liver glycogen, reduce lactate dehydrogenase and creatine kinase extravasation, and protect muscle tissue from damage in the endurance swimming mice. HFOs-AK were shown to enhance Na^+^-K^+^-ATPase and Ca^2+^-Mg^2+^-ATPase activities and increase ATP content in muscle tissue. Meanwhile, HFOs-AK also showed significantly antioxidant ability by increasing the activities of superoxide dismutase and glutathione peroxidase in the liver and decreasing the level of malondialdehyde. Further studies showed that HFOs-AK could regulate the body’s energy metabolism and thus exert its anti-fatigue effects by activating the AMPK signaling pathway and up-regulating the expression of p-AMPK and PGC-α proteins. Therefore, HFOs-AK can be used as an auxiliary functional dietary molecules to exert its good anti-fatigue activity and be applied to anti-fatigue functional foods.

## 1. Introduction

Marine resources are used as a source of various health foods, and protein hydrolysates and peptides extracted from marine organisms have a variety of bioactive functions as well as pharmaceutical effects, like metal-chelating, antioxidant, anti-inflammatory, anti-hypertensive, antimicrobial, anticancer, and immunomodulatory activities [1,2]. Bioactive peptides are specific small amino acid fragments (usually 3 to 20 amino acids) obtained from natural sources that are capable of causing physicochemical changes in normal bodily processes, and these physicochemical effects arise from their unique amino acid composition, sequence, and molecular weights [3,4]. Peptides typically comprise a variety of amino acids, among which branched-chain amino acids (BCAAs) and aromatic amino acids (AAAs) are present in a molar ratio known as the Fischer ratio (or F-value). High Fischer ratio oligopeptides (HFOs) are mixed small-molecule peptides with a BCAA/AAA ratio greater than 20, whose oligopeptides have molecular weights between 200 and 1000 Da and are composed of 2 to 9 amino acids [5]. Thanks to their distinct amino acid and peptide makeup, HFO exhibit a range of impressive biological properties, such as serving as an adjunct therapy for hepatic encephalopathy [6], sobering and anti-intoxication activity [7], antioxidant activity [5,8], adjunctive therapy for phenylketonuria [9], anti-fatigue activity [10], and some clinical surgical treatments [11]. Every year, low-valuable protein resources from both land and sea go untapped in significant quantities. Transforming these resources into bioactive oligopeptides, marked by an HFO, promises substantial economic, nutritional, and medical benefits. Therefore, HFO have received widespread attention, and there have been successful cases ranging from hard-shell mussels [12], whey proteins [13], corn [14], Antarctic krill [5], flaxseed [15,16], gluten [17], goat’s milk whey protein [18], bonito [7], oyster (*Pinctada martensii*) meat [19], and *Aspergillus niger* [20]. Hence, HFOs hold great promise for practical use in clinical settings because of their impressive bioactive properties. Comprehensive research into the physiological functions and underlying mechanisms of HFOs is crucial in unlocking their potential for integration into pharmaceutical and health-enhancing products.

Fatigue is usually divided into physiological or pathological causes which can lead to various discomforts and are related to various diseases. However, some studies have shown that exercise leads to fatigue, and the fatigue caused by high-intensity exercise is related to injury, the working ability and liver glycogen level, resulting in energy source [21]. Concurrently, the buildup of blood lactic acid (BLA) and blood urea nitrogen (BUN) while exercising results in the accumulation and threshold of metabolites, culminating in physical exhaustion [22]. During physical activity, the body extensively utilizes energy storage molecules, such as adenosine triphosphate (ATP), glucose, and fat. This depletion ultimately results in diminished skeletal muscle function, rendering individuals unable to sustain the intended exercise intensity. Consequently, this can lead to injury and increases in creatine kinase (CK) level [23]. In recent years, some related studies have been carried out to find natural substances with anti-fatigue activity to avoid the damage caused by ingesting chemical drugs, and these investigations have shown that the active peptides extracted from natural foods are safe and effective in preventing and relieving fatigue. For example, Corn peptides (CPs) are rich in amino acids, including glutamic acid, so that it has a significant ability to relieve exercise-induced fatigue. Moreover, they have significant anti-fatigue properties that help mitigate damage caused by synthetic drugs [21]. Soybean peptides, known as FSPPs, play a crucial role in facilitating protein synthesis and contributing to the energy provision for cells within skeletal muscle. Consequently, they trigger an anti-fatigue response [24]. On the other hand, the anti-fatigue properties of sea cucumber peptide (SCP) are attributed to its ability to restore normal energy metabolism while mitigating oxidative stress and inflammatory responses [23]. Moreover, oligopeptides with a high F value have a unique amino acid composition, which can increases the AAA content of the body’s blood [12]. Therefore, compared with normal oligopeptides, HFO may have better anti-fatigue effects because of their unique amino acid composition, so as to develop anti-fatigue functional foods. However, the anti-fatigue mechanism of HFO still needs to be studied systematically.

Antarctic krill (*Euphausia superba*), which mainly lives in Antarctica, is the world’s richest animal protein resource, with high nutritional value [25]. Antarctic krill protein contains all the essential amino acids required by the human body, and its bioactivity was evaluated to be higher than that of other animal and plant proteins and dairy proteins (casein) [26], so peptides with significant bioactivities were prepared from hydrolysates of Antarctic krill protein [27]. For example, Antarctic krill peptides not only significantly attenuates CCl_4_-induced hepatic injury by activating the Nrf2/HO-1 pathway [28], but also ameliorates hepatic fibrosis by improving gut microbiota-mediated bile acid-NLRP3 pathway [29]. According to behavioral experiments, SSDAFFPFR and SNVDFMF from Antarctic krill can ameliorate scopolamine-induced memory deficits by altering the behavior of mice [30]. Ca-EEEFDATR chelate has effects on MC3T3-E1 cells with osteoblast proliferation, differentiation, and mineralization [31]. Peptides of VW and LKY have antihypertensive effects in spontaneously hypertensive rats [32]. In our previous research, HFO from Antarctic krill (HFOs-AK) was enzymatically prepared with alcalase and flavorzyme, and it showcased promising antioxidant properties in vitro [5]. However, more in-depth functional evaluation and mechanism studies on HFOs-AK, especially the anti-fatigue function closely related to oxidative stress, are still lacking. Hence, the aim of this research was to methodically assess the effectiveness of HFOs-AK in combating fatigue and delve into its mechanism of operation by establishing a vigorous swimming-induced fatigue model in mice. This study provides experimental evidence and a theoretical basis for the application of Antarctic krill oligopeptides in fatigue reduction related functional foods.

## 2. Results

### 2.1. Effect of HFOs-AK on Body Weight and Organ Index of Fatigue Model of Mice

As depicted in Table 1, following gavage for 30 days, mice increased their body weight in all groups, but there was no significant difference in the body weight of mice among the blank, model, positive control, and HFOs-AK groups. It demonstrates that gavage of HFOs-AK does not negatively affect the growth and development of mice, and the mice in all groups were in a normal growth and feeding environment.

According to the organ indices expressed in Table 2, the coefficients of the liver, spleen, kidney, and thymus of mice in the low-, medium-, and high-dose groups of HFOs-AK were not significantly different (*p* > 0.05) compared to those of the model group. Experimental data indicated that gavage of HFOs-AK did not produce adverse effects on the growth of mice.

### 2.2. Effect of HFOs-AK on Tissue Morphology of Fatigue Model of Mice

**Figure 1:**
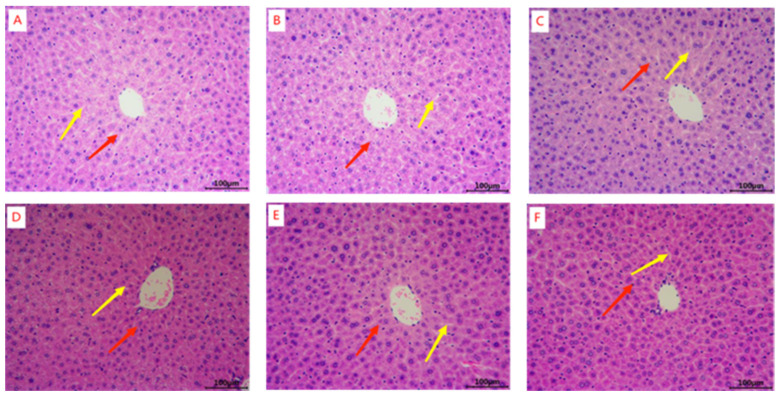
H&E staining of mouse liver tissue samples. The fatigue model of mice was established by an endurance swimming method. Whey peptides (0.5 mg/g·bw/d) served as a positive control. (**A**) Blank; (**B**) Model; (**C**) Whey peptides (0.5 mg/g·bw/d); (**D**) Low-dose of HFOs-AK (0.1 mg/g·bw/d); (**E**) Mid-dose of HFOs-AK (0.3 mg/g·bw/d); (**F**) High-dose of HFOs-AK (0.5 mg/g·bw/d). The yellow arrow indicates the hepatic sinusoids, and the red arrow indicates the hepatic cord.

The liver nuclei of mice in the blank group (Figure 1A) appeared intact and the hepatocytes showed no signs of necrosis in the H&E staining results. The hepatic blood sinusoids (yellow arrows) and hepatic cords (red arrowheads) were neatly aligned, and the hepatic lobules were clear in outline. Compared with the blank control group, the structure of the liver lobules in the model group (Figure 1B), positive control group (Figure 1C), and HFOs-AK groups (Figure 1D–F) was in a normal state, and the arrangement of the hepatic blood sinusoids (yellow arrowheads) and the hepatic cords (red arrowheads) did not undergo any alteration of the pathological state. It indicates that HFOs-AK do not cause damage to the mouse liver.

**Figure 2:**
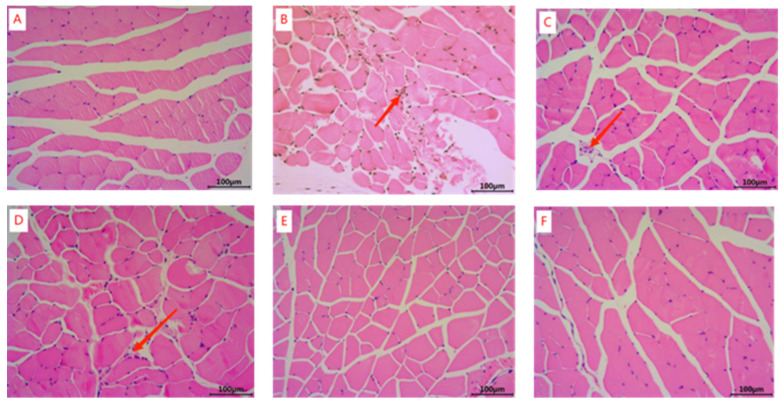
H&E staining of mouse muscle tissue samples. Whey peptides (0.5 g/g·bw/d) served as a positive control. (**A**) Blank; (**B**) Model; (**C**) Whey peptides (0.5 mg/g·bw/d); (**D**) Low-dose of HFOs-AK (0.1 mg/g·bw/d); (**E**) Mid-dose of HFOs-AK (0.3 mg/g·bw/d); (**F**) High-dose of HFOs-AK (0.5 mg/g·bw/d). The red arrows in the diagram indicate damage and irregular alignment of the gastrocnemius muscles of mice.

The results of H&E staining of muscle tissues are shown in Figure 2. The muscle fibers of the gastrocnemius muscle of mice in the blank group (Figure 2A) were uniformly aligned with clear transverse striations. Compared with the blank group, the gastrocnemius muscles of mice in the model group (Figure 2B) showed damage (red arrow) and irregular arrangement. Compared with the model group, the low-, medium-, and high-dose HFOs-AK groups of HFOs-AK (Figure 2D–F) showed a reduction in the degree of damage appearing in the fibers of the mouse gastrocnemius muscle, indicating that HFOs-AK could effectively ameliorate the muscle damage in mice.

### 2.3. Effect of HFOs-AK on Exercise Capacity and Metabolite Levels of BUN, Lactic Acid (LA), and Blood Ammonia (BA) in Fatigue Model of Mice

**Figure 3:**
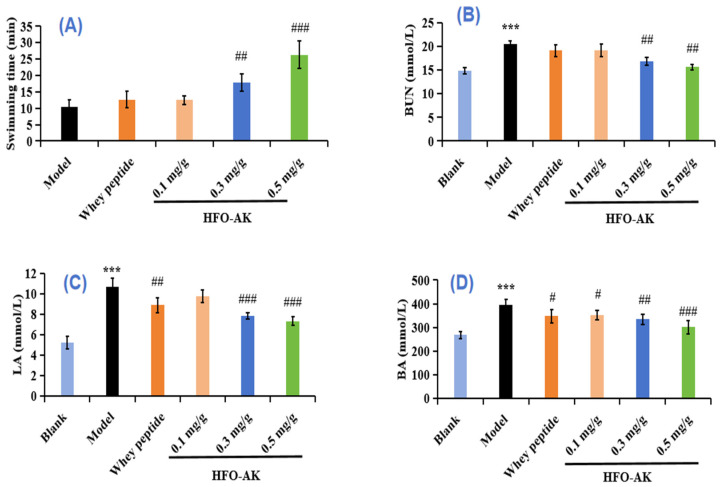
Effect of HFOs-AK on exercise capacity (**A**) and in vivo metabolites of BUN (**B**), LA (**C**), and BA (**D**) in fatigue model of mice. Whey peptides (0.5 g/g·bw/d) served as a positive control. *** *p* < 0.001 compared to blank control group. ^###^ *p* < 0.001, ^##^ *p* < 0.01 and ^#^ *p* < 0.05 compared to model group.

Fatigue is the feeling of exhaustion that sets in following extended or intense physical activity, indicating a decline in physiological capacity and the body’s ability to sustain its functions [10], so it can be measured by increased exercise endurance to measure the effect of fatigue resistance [23]. The effect of HFOs-AK on the exercise capacity of fatigue model of mice is shown in Figure 3A. The model group showed a time to exhaustion of 10.35 min, but the time to exhaustion was prolonged in all the HFOs-AK-administered groups by 20.64 ± 13.05% (low), 72.84 ± 25.63% (medium), and 154.14 ± 40.34% (high). In addition, there was a significant difference between the medium (17.89 ± 2.65 min) and high (26.31 ± 4.18 min-dose HFOs-AK groups compared to the model group (*p* < 0.01). Also, as the dose of HFOs-AK was increased, the endurance swimming time of the mice increased, showing a dose-dependent pattern. These data suggest that HFOs-AK have an anti-fatigue effect on mice, and the higher the dose, the more obvious the effect.

Metabolic waste is negatively correlated with exercise endurance [33]. When the energy supply in the body is insufficient, the energy needed by the body can be provided by protein metabolism. The process of protein catabolism produces large amounts of NH_3_ and CO_2_, and these metabolic wastes are synthesized into urea in the liver of the organism, which is excreted via the humoral circulation. The effect of HFOs-AK on BUN in the fatigue model of mice is shown in Figure 3B. Compared with the blank group (14.85 ± 0.71 mmol/L), the model group of mice, which had been subjected to swimming and had not been gavaged with HFOs-AK, had the highest BUN levels of 20.49 ± 0.71 mmol/L, indicating successful modeling of low exercise tolerance in mice. The BUN levels in low-, medium-, and high-dose HFOs-AK groups were dose-dependently decreased by 6.74% ± 6.55%, 17.68% ± 3.82%, and 23.85% ± 2.93%, respectively. Moreover, the BUN level in the high-dose HFOs-AK group of HFOs-AK was 15.60 ± 0.60 mmol/L, which was highly significant (*p* < 0.001) compared with the model group. It indicates that HFOs-AK participate in the process of collective energy metabolism and reduce the original protein metabolism to effectively reduce the BUN content in the body.

Glycolysis is the main source of energy for high-intensity exercise in a short period of time. Lactic acid (LA) is produced under anaerobic conditions by glycolysis. As glycolysis accelerates, LA increases in muscle and accumulates in the body, lowering the pH of the body and leading to fatigue as well as reduced exercise capacity [34]. The effects of HFOs-AK on LA in fatigue model of mice are shown in Figure 3C. After endurance swimming exercise in mice, the LA levels in low, medium and high-dose HFOs-AK groups were 9.74 ± 0.61, 7.83 ± 0.34, and 7.32 ± 0.43 mmol/L, respectively, which were significantly decreased (*p* < 0.001) compared to the model group (10.67 ± 0.87 mmol/L). It shows that HFOs-AK have certain anti-fatigue activity by reducing the accumulation of LA in the muscle and body of mice.

Adenosine triphosphate (ATP) is the direct source of energy for all vital activities in the body, and nutrients, such as creatine phosphate, glycogen, glucose, fat, and protein, provide indirect energy for exercise [35]. When proteins and amino acids are degraded to provide energy for the body, a large amount of ammonia enters the bloodstream, and the high concentration of NH_3_ in the body affects the energy metabolism and motor balance of the body, causing fatigue [36]. The effects of HFOs-AK on blood ammonia (BA) in the fatigue model of mice are shown in Figure 3D. Compared with the model group (395.19 ± 22.41 mmol/L), the BA contents in the low-dose HFOs-AK (352.09 ± 19.76 mmol/L), medium-dose HFOs-AK (334.46 ± 21.08 mmol/L), high-dose HFOs-AK (301.81 ± 27.66 mmol/L), and the positive control (347.52 ± 28.72 mmol/L) groups were significantly decreased (*p* < 0.001). It indicates that HFOs-AK play its anti-fatigue activity through reducing the energy metabolism and exercise homeostasis of the body.

### 2.4. Effect of HFOs-AK on Lactate Dehydrogenase (LDH) and CK of Fatigue Model of Mice

**Figure 4:**
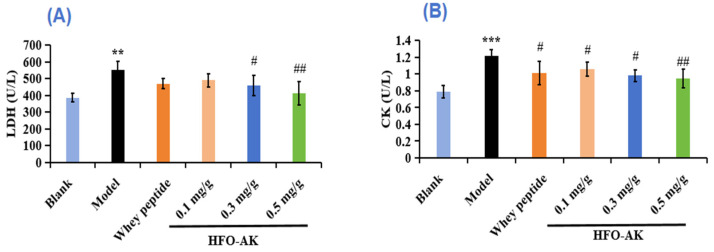
Effect of HFOs-AK on LDH (**A**) and CK (**B**) activity in fatigue model of mice. Whey peptides (0.5 g/g·bw/d) served as a positive control. *** *p* < 0.001 and ** *p* < 0.01 compared to blank control group. ^##^ *p* < 0.01 and ^#^ *p* < 0.05 compared to model group.

LDH mainly catalyzes the mutual conversion between acetone and LA, indicating the degree of lactate metabolism. Increases in LDH concentrations caused by high-intensity exercise can lead to muscle damage. Therefore, LDH is also considered to be one of the specific markers for assessing physical fatigue [37]. The effects of HFOs-AK on LDH in fatigue mice are shown in Figure 4A. LDH levels in the low, medium and high-dose HFOs-AK groups were 490.30 ± 40.28, 458.08 ± 60.21, and 412.35 ± 69.76 U/L, respectively, significantly lower than that in the model group (552.53 ± 50.74 U/L). It suggests that HFOs-AK can alleviate exercise fatigue and protect muscles by reducing LDH content.

CK, mainly found in the cytoplasm and mitochondria, is an important kinase and involved in intracellular energy conversion, ATP production, and muscle contraction. Serum CK is mainly derived from skeletal muscle in the organism; therefore, changes in serum CK activity can be used as an indicator to assess skeletal muscle injury and recovery after exercise in the organism, and the higher the CK activity, the more severe the skeletal muscle injury and fatigue [37]. The effect of HFOs-AK on CK in fatigue mice is shown in Figure 4B. Compared with the blank group (0.79 ± 0.08 U/L), the CK activity in the model group (1.22 ± 0.07 U/L) was significantly increased, indicating successful modeling of fatigued mice. However, the CK levels in the low-, medium-, and high dose groups of HFOs-AK were 1.06 ± 0.08, 0.98 ± 0.07, and 0.94 ± 0.11 U/L, respectively, and were reduced by 12.89% ± 6.82%, 19.48% ± 5.74%, and 22.50% ± 9.01%, respectively, in comparison with the model group. These results indicate that HFOs-AK are able to attenuate muscle damage caused by strenuous exercise.

### 2.5. Effect of HFOs-AK on Blood Glucose (BG), Muscle Glycogen (MG), and Liver Glycogen (LG) of Fatigue Model of Mice

Glycogen are used for long-term energy storage, which can be rapidly consumed when fatigue occurs to meet the urgent need for glucose, and increased glycogen levels in the liver and muscles can enhance endurance during intense exercise. During strenuous exercise, the organism enhances glycogen metabolism and lowers BG levels to maintain them in the normal physiological range [37]. Therefore, glycogen content is an important test indicator for fatigue resistance [38].

**Figure 5:**
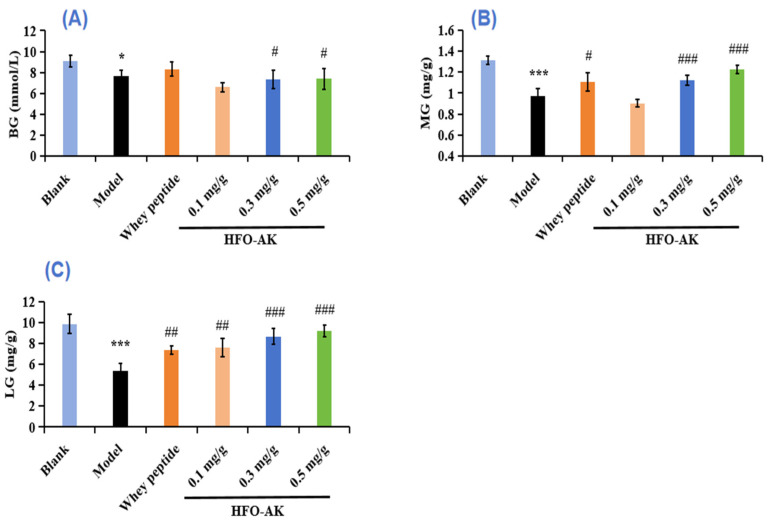
Effect of HFOs-AK on the contents of BG (**A**), MG (**B**), and LG (**C**) in fatigue model of mice. Whey peptides (0.5 g/g·bw/d) served as a positive control. *** *p* < 0.001 and * *p* < 0.05 compared to blank control group. ^###^ *p* < 0.001, ^##^ *p* < 0.01 and ^#^ *p* < 0.05 compared to model group.

As shown in Figure 5A, the level of BG in model group (7.69 ± 0.51 mmol/L) was significantly lower than that in the blank group (9.06 ± 0.56 mmol/L) (*p* < 0.05). Furthermore, the medium (7.33 ± 0.90 mmol/L) and high (7.39 ± 1.00 mmol/L-dosage groups of HFOs-AK exhibited notably elevated blood glucose levels compared to the model group (*p* < 0.05). This suggests that HFOs-AK have the potential to enhance blood glucose utilization and modulate energy metabolism within the exhausted mice’s bodies, which could improve the organism’s exercise tolerance.

Figure 5B,C showed the effect of HFOs-AK on MG and LG levels in the fatigue mice. The data indicated that the MG levels of the HFOs-AK groups, especially the medium-dose group (1.12 ± 0.05 mg/g) and the high-dose group (1.22 ± 0.04 mg/g), were significantly higher (0.97 ± 0.07 mg/g) than that model group (*p* < 0.001) (Figure 5B).

The results in Figure 5C showed that the LG level of the model group (5.40 ± 0.67 mg/g) was significantly lower (*p* < 0.001) than that (9.85 ± 0.89 mg/g) of the blank group. Treatment with HFOs-AK, the LG levels of fatigue mice in the low, medium and high-dose HFOs-AK groups were 7.58 ± 0.89, 8.66 ± 0.78, and 9.21 ± 0.55 mg/g, respectively. The findings revealed markedly elevated levels of MG and LG in the experimental group compared to those in the control group (*p* < 0.01). Consequently, as the dosage increased, there was a notable rise in the average concentrations of MG and LG within the mouse system (*p* < 0.05). In conclusion, gavage of HFOs-AK can increase glycogen content, regulate energy metabolism, and enhance exercise tolerance in mice.

### 2.6. Effect of HFOs-AK on ATP Content and Activities of Na^+^-K^+^-ATPase and Ca^2+^-Mg^2+^-ATPase of Fatigue Model of Mice

**Figure 6:**
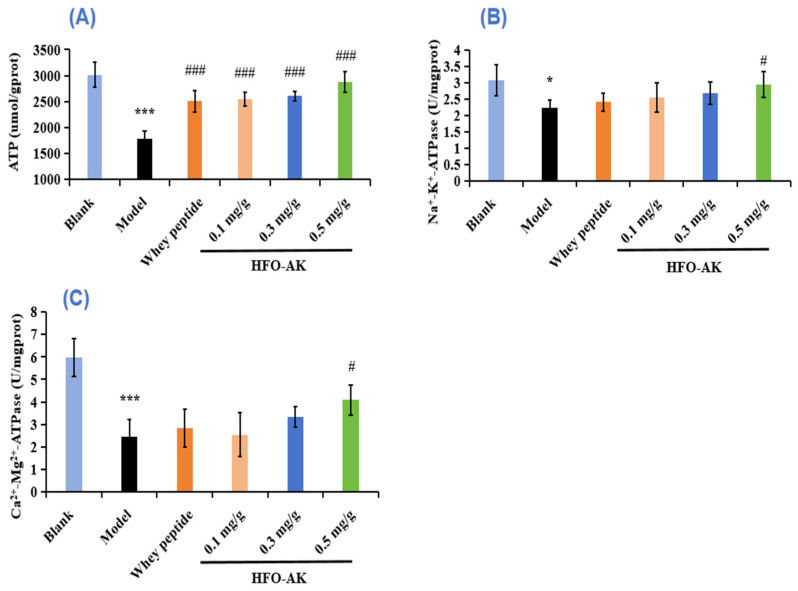
Effect of HFOs-AK on ATP content (**A**), Na^+^-K^+^-ATPase (**B**) and Ca^2+^-Mg^2+^-ATPase (**C**) activities in fatigue mice. Whey peptides (0.5 g/g·bw/d) served as a positive control. *** *p* < 0.001 and * *p* < 0.05 compared to blank control group. ^###^ *p* < 0.001 and ^#^ *p* < 0.05 compared to model group.

The most basic carrier of energy conversion in an organism is ATP. Usually, ATP levels decrease when the cell is apoptotic, necrotic, or in some pathological state, which indicates that the mitochondria have impaired functionality with decreased viability. The impact of HFOs-AK on ATP levels in fatigued mice is illustrated in Figure 6A. In comparison to the control group (3013.48 ± 241.92 μmol/g prot), mice in the model group (1785.31 ± 148.19 μmol/g prot) exhibited a notable decline in ATP content (*p* < 0.001). Conversely, mice administered with low, medium, and high doses of HFOs-AK demonstrated ATP levels of 2546.10 ± 125.33, 2606.62 ± 87.05, and 2879.27 ± 197.19 μmol/g prot, respectively, indicating a significant rise and a marked disparity in comparison to the model group (*p* < 0.001). These findings suggest that HFOs-AK directly enhance energy provision in vivo, thereby sustaining organismal energy metabolism and enhancing the fatigue resistance of the mice.

Na^+^-K^+^-ATPase and Ca^2+^-Mg^2+^-ATPase play crucial roles in the physiological processes of material transfer, energy conversion, and information transfer, and are the key enzymes in degrading ATP [39]. When their activity decreases, the level of reactive oxygen species (ROS) in the mitochondria increases, which results in impaired mitochondrial function and impaired energy synthesis, producing fatigue. Figure 6B,C showed the effect of HFOs-AK on Na^+^-K^+^-ATPase and Ca^2+^-Mg^2+^-ATPase activity in fatigue mice. After endurance swimming, the Na^+^-K^+^-ATPase activity in mice decreased from 3.06 ± 0.47 U/mg prot to 2.22 ± 0.24 U/mg prot, but the Na^+^-K^+^-ATPase activity was significantly increased to 2.94 ± 0.39 U/mg prot after administration of 0.5 mg/g HFOs-AK (*p* < 0.001) (Figure 6A). The Na^+^-K^+^-ATPase activity showed the similar trend. The Ca^2+^-Mg^2+^-ATPase decreased from 5.97 ± 0.85 U/mg prot to 2.45 ± 0.77 U/mg prot in mice after endurance swimming, but the Ca^2+^-Mg^2+^-ATPase activity was significantly increased to 4.08 ± 0.66 U/mg prot after administration of 0.5 mg/g HFOs-AK (*p* < 0.001) (Figure 6C). Therefore, the administration of HFOs-AK can improve mitochondrial function, enhance the breakdown of ATP in skeletal muscle cells, fuel muscle cells with energy, and enhance the body’s capacity to combat fatigue.

### 2.7. Effect of HFOs-AK on Antioxidant Capacity of Fatigue Model of Mice

It has been shown that superfluous ROS are produced during strenuous exercise, which can lead to fatigue and lipid peroxidation damage. Antioxidant enzymes like superoxide dismutase (SOD) and glutathione peroxidase (GSH-Px) play a key role in eliminating surplus ROS produced during intense physical activity in order to avert oxidative harm, thereby sidestepping cellular injury and muscle depletion [40,41].

**Figure 7:**
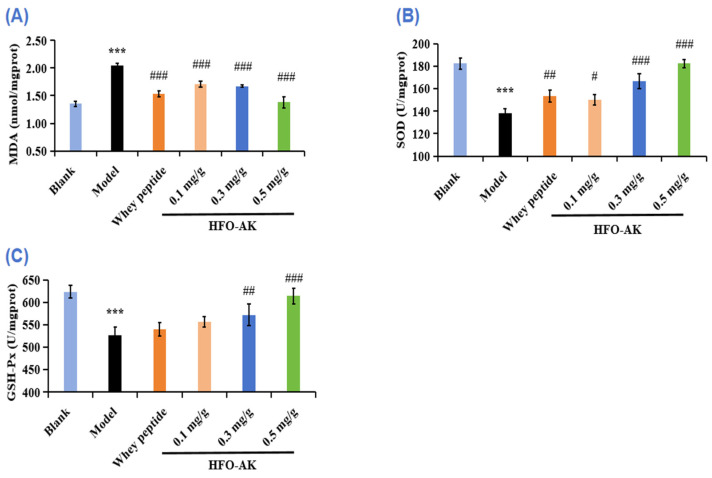
Effect of HFOs-AK on MDA (**A**) content, and SOD (**B**) and GSH-Px (**C**) activities in fatigue model of mice. Whey peptides (0.5 g/g·bw/d) served as a positive control. *** *p* < 0.001 compared to blank control group. ^###^ *p* < 0.001, ^##^ *p* < 0.01 and ^#^ *p* < 0.05 compared to model group.

Malondialdehyde (MDA), a byproduct of cell membrane breakdown due to lipid peroxidation, increases as fatigue intensifies. Figure 7A illustrates a significant rise in MDA levels in fatigued mice, jumping from 1.36 ± 0.05 nmol/mg prot to 2.05 ± 0.03 nmol/mg prot post exhaustive swimming. Comparatively, the MDA concentrations in groups administered with varying doses of HFOs-AK were notably decreased by 16.76% ± 2.56%, 18.35% ± 1.08%, and 32.53% ± 5.04%, respectively (*p* < 0.05) when contrasted with the control group. These findings suggest that HFOs-AK have the potential to diminish liver injury by alleviating oxidative stress, as evidenced by reduced MDA formation in the livers of fatigued mice.

SOD is an antioxidant enzyme that helps to improve cellular resistance to oxidative stress. Figure 7B represents the effect of HFOs-AK on SOD activity in fatigue mice. According to the data, the SOD activity in the model group (138.51 ± 3.62 U/mg prot) is significantly lower compared to the blank group (182.45 ± 4.74 U/mg prot). Furthermore, in the medium- and high-dose groups of HFOs-AK, there was a noticeable increase in SOD activity in the liver tissue of fatigued mice, with increments of 20.36% ± 4.62% and 31.71% ± 2.61%, respectively, significantly higher than that of the model group. Additionally, the SOD activity in the medium- (166.71 ± 6.39 U/mg prot) and high-dose groups (182.43 ± 3.62 U/mg prot) of HFOs-AK is slightly higher than that of the positive control group (153.49 ± 5.25 U/mg prot). This indicates that HFOs-AK have a protective effect on the mouse liver, and this effect is dose-dependent.

GSH-Px is a widely recognized antioxidant known for its ability to combat ROS and is frequently utilized as a biomarker for gauging the level of oxidative stress-induced damage. According to the data presented in Figure 7C, the concentration of GSH-Px in the model group presented a noteworthy reduction to 526.67 ± 18.04 U/mg prot, a figure significantly below that of the control group (623.20 ± 14.25 U/mg prot) (*p* < 0.001). In addition, the GSH-Px levels in the medium- and high dosage of HFOs-AK groups stood at 572 ± 23.78 and 614 ± 17.74 U/mg prot, respectively, while the GSH-Px content in the high dosage of HFOs-AK group was significantly elevated compared to that of the model group (*p* < 0.001). These findings indicated that HFOs-AK was able to increase GSH-Px levels in liver tissues of fatigue mice, suggesting that HFOs-AK could increase liver antioxidant levels and reduce injury.

In summary, referring to the related literatures [42], it could be theorized that HFOs-AK could help alleviate fatigue by enhancing the functioning of antioxidant enzymes within the body and limiting the generation of lipid peroxides in cellular membranes.

### 2.8. Effect of HFOs-AK on Protein Expression of the AMPK/PGC-1α/Nrf2 Signaling Pathways in Fatigue Model of Mice

Mitochondria are pivotal in energy metabolism, with mitochondrial dysfunction and structural irregularities being potential culprits behind fatigue. AMP-activated protein kinase (AMPK) is instrumental in regulating glucose and lipid metabolism, aiding in the maintenance of ATP levels across diverse conditions. The AMPK signaling pathway is triggered during sustained physical activity, enhancing basal glucose uptake. Peroxisome proliferator-activated receptor gamma coactivator-1 alpha (PGC-1α) emerges as a vital player in mitochondrial biogenesis and other physiological functions, predominantly observed in skeletal muscle tissues rich in mitochondria [43,44]. AMPK-1α and PGC-1α are key regulators of metabolism, and they increase resistance mainly by increasing the degradation of glucogenesis, glucose enzymes, sugar intake, and fatty acid oxidation. During exercise, AMPK is activated and electro-stimulated to shrink, thereby promoting the intake of basic glucose. In our study, HFOs-AK was found to enhance the expression of the phosphorus form of AMPK in the liver and bone muscle of mouse motors through analysis of protein traces. In addition, we also found an enhancement expression of PGC-1α (transcript co-activating factor), which activates the metabolic and biological processes in fibroblasts, thereby converting type II muscle fibers into type I muscle fiber. They can use oxygen and synthesize more ATP for continuous muscle contractions and become more fatigued.

**Figure 8:**
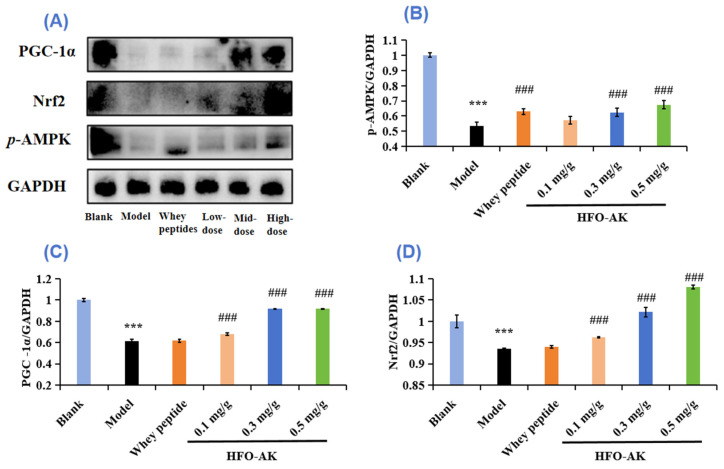
Protein map of HFOs-AK on anti-fatigue signaling pathway in mice (**A**). Effect of HFOs-AK on expression levels of *p*-AMPK (**B**), PGC-1α (**C**), and Nrf2 (**D**) protein in mice. Whey peptides (0.5 g/g·bw/d) served as a positive control. *** *p* < 0.001 compared to blank control group. ^###^ *p* < 0.001 compared to model group.

Figure 8B shows the effect of HFOs-AK on the *p*-AMPK protein expression level in fatigue mice. The *p*-AMPK protein expression level in the model group (0.53 ± 0.03 GAPDH) was significantly reduced (*p* < 0.001) compared with the blank group. The study revealed a notable increase (*p* < 0.001) in the *p*-AMPK protein levels in the liver tissue of mice treated with 0.3 and 0.5 mg/g of HFOs-AK, elevating them to 0.62 ± 0.03 GAPDH and 0.67 ± 0.03 GAPDH, respectively. These findings indicate that HFOs-AK play a role in modulating energy metabolism and demonstrating anti-fatigue properties by activating the AMPK pathway.

Figure 8C shows the effect of HFOs-AK on the protein expression level of PGC-1α in fatigue mice. Compared with the blank group, the expression level of the PGC-1α protein was measured at 0.61 ± 0.02 GAPDH in the control group and showed a significant decrease (*p* < 0.001). In contrast, the liver tissue of the mouse model exhibited a noteworthy increase in PGC-1α protein levels to 0.92 ± 0.002 GAPDH and 0.92 ± 0.003 GAPDH after being treated with 0.3 and 0.5 mg/g of HFOs-AK, respectively. These findings indicate that HFOs-AK can influence energy metabolism and reduce fatigue by stimulating the PGC-1α pathway.

Mitochondria and other organelles of skeletal muscle and liver are vulnerable to lipid peroxidation [45]. During intense swimming sessions, excessive ROS are generated and build up significantly in the mouse’s body. This accumulation leads to harmful effects by attacking crucial organic molecules and cell structures, causing oxidative stress and the creation of lipid peroxidation byproducts like MDA [46]. The nuclear factor E2-related factor 2 (Nrf2) signaling pathway serves as a crucial target for combating fatigue and various diseases associated with oxidative stress [45]. This pathway primarily operates by triggering the production of phase II enzymes that aid in defending against oxidative stress and scavenging ROS. In essence, Nrf2 acts as a pivotal regulator of cellular oxidation on a transcriptional level, controlling the expression of key downstream factors [47]. The impact of HFOs-AK on Nrf2 protein expression levels in fatigued mice, as depicted in Figure 8D, is evident. In comparison to the control group, the model group exhibited a noteworthy decrease in Nrf2 protein expression levels (0.94 ± 0.022 GAPDH) (*p* < 0.001). Conversely, the Nrf2 protein levels in liver tissues of fatigued mice significantly increased (*p* < 0.001) to 0.96 ± 0.002, 1.02 ± 0.011, and 1.08 ± 0.005 GAPDH following the administration of 0.1, 0.3, and 0.5 mg/g HFOs-AK, respectively. Subsequently, HFOs-AK modulates energy metabolism and exerts anti-fatigue effects by activating the Nrf2 pathway.

## 3. Discussion

Recent research has demonstrated the vital role of branched-chain amino acids in HFO as essential nutrients for tissue synthesis, energy provision, and overall health. Specifically, leucine and isoleucine undergo transamination to acetyl coenzyme A (acetyl-CoA), which enters the citric acid cycle, enhancing energy production in active muscles. On the other hand, isoleucine and valine are transformed into α-keto acid through transamination, further metabolized into succinyl coenzyme A, malate, and pyruvate, and eventually converting into alanine. This alanine is then transported to the liver via the bloodstream, where it is converted into pyruvate and subsequently into glucose. This glucose is carried back to the muscle for energy utilization during physical exertion [48]. The addition of HFOs-AK supplementation, along with swimming training, has been shown to notably enhance glycogen storage in both the liver and muscle. As demonstrated in Figure 3A, these adaptations led to a considerable enhancement in exercise endurance performance, an extension of the duration prior to exhaustion during physical activity in mice, and the effective preservation of glycogen levels following swimming exercise [49].

Maintaining stable blood glucose levels is essential for the proper functioning of organs, as blood glucose serves as a primary energy source for the central nervous system and red blood cells. Vigorous physical activity over an extended period depletes carbohydrate stores and lowers blood glucose levels, typically stored as glycogen in the liver and muscles. Research indicates that glycogen stored in these areas serves as the primary energy source during prolonged endurance exercise of moderate to high intensity [50]. Simultaneously, intense physical activity results in a significant energy expenditure and the production of surplus free radicals in muscle tissue. The excessive buildup of these free radicals triggers lipid peroxidation in the cell membrane, altering the normal composition of cellular proteins and genetic material. Consequently, this disrupts the permeability of the mitochondria’s internal membrane, leading to oxidative fatigue. Bioactive peptides have the ability to neutralize free radicals within lipids or impede the propagation of free radical chains, safeguarding cells against oxidative harm. This process enhances the body’s antioxidant capabilities and boosts its resistance to fatigue. It has been found that a large number of free radicals are produced during exhaustive swimming, leading to fatigue, and are prone to lipid peroxidation damage to organelles such as mitochondria in liver and skeletal muscle tissue [45].

Among natural active substances, bioactive peptides have received attention for their ability to reduce fatigue-induced muscle damage to varying degrees. Short peptides extracted from deer blood were evaluated in swimming mice and not only increased hepatic glycogen (HG) stores to improve exercise endurance, but also helped to reduce muscle lactate (MLA) accumulation and HG increase, exerting anti-fatigue activity [51]. Supplementation with sea dragon peptides exerted anti-fatigue effects on exercise-fatigued mice, and its mechanism may be to inhibit oxidative stress, improve grip strength, and prolong the swimming time of mice by reducing the accumulation of metabolites LA, BUN, and MDA. It promotes the expression of proteins related to the AMPK/PGC-1α and Nrf2 signaling pathways and regulates gut microbial homeostasis [52]. Using a mouse model of endurance swimming, peptides from striped bass (*Trichiurus lepturus*) [22], tilapia (*Oreochromis nilotica* L.) [53], yak bone [54], hemp seed [15], ankylosing fish [55], seahorses [50], and sea cucumbers [23] can prolong endurance swimming time in mice, reduce metabolites and antioxidant activity in vivo, and increase glycogen reserves and energy metabolism while decreasing the levels of LDH and CK due to muscular fatigue, resulting in anti-fatigue effects.

**Figure 9:**
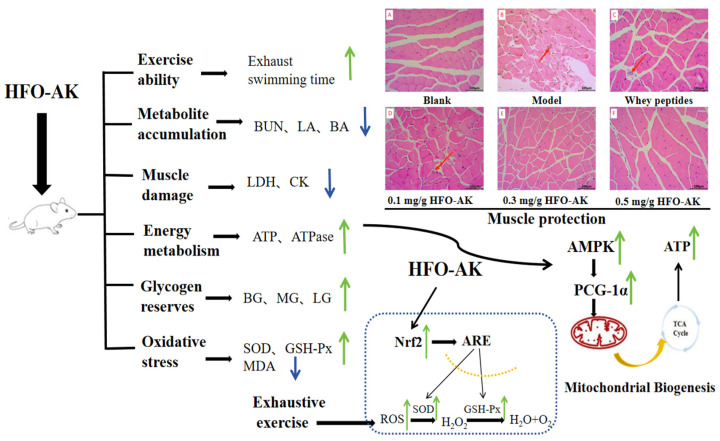
Anti-fatigue mechanisms of HFOs-AK on fatigue model of mice. The blue arrows in the chart indicate that the data is trending downward. The green arrows in the chart indicate that the data is trending upwards. The red arrows in the diagram indicate damage and irregular alignment of the gastrocnemius muscles of mice.

The Figure 9 show that when the energy ATP supply in the body is insufficient, the levels of key enzymes, including Na^+^-K^+^-ATPase and Ca^2+^-Mg^2+^-ATPase, which degrade ATP, will also decrease. The result is that the contents of BUN, LA, and BA are decreased, while the levels of BG, MG, and LG will continue to increase. While the muscles will produce large amounts of LDH and CK, which will lead to muscle damage. An overabundance of ROS can trigger oxidative stress, thereby inhibiting the function of internal antioxidant enzymes like SOD, and GSH-Px.

Typically, AMPK boosts the oxidation of fatty acids by inhibiting the formation of malonyl coenzyme A. Yet, during prolonged activation of AMPK, it governs the oxidation of fatty acids differently by activating peroxisome proliferator-activated Receptor α (PPARα) and PGC-1α. Additionally, it controls the uptake of glucose and the synthesis of mitochondria from scratch, which relies entirely on PGC-1α, whether in a living organism or in a laboratory setting [56]. The signaling cascade reactions of AMPK/PGC-1α are crucial across mammalian species, spanning from mice to humans, particularly in the skeletal muscles, where they contribute significantly to mitochondrial biosynthesis and maturation [57]. In the realm of muscle physiology, PGC-1α emerges as a pivotal player, igniting the process of mitochondrial biogenesis. This, in turn, bolsters both the quantity and quality of mitochondria within skeletal muscle fibers. Notably, skeletal muscle tissue showcases abundant expression of PGC-1α, orchestrating the synthesis of ATP to stave off muscle dysfunction. However, when PGC1-α veers off its typical course, metabolic disorders of an associative nature ensue. AMPK, in its active state, assumes a crucial role in this scenario, directly modulating the activity of PGC-1α through phosphorylation [58]. Thus, the tandem action of AMPK and PGC-1α emerges as the linchpin of the body’s resilience against fatigue. In Figure 9, it is evident that there was a notable increase in the levels of AMPK/PGC-1α proteins in the liver tissue of tired mice following the administration of HFOs-AK (*p* < 0.001). The mechanism suggests that HFOs-AK can activate the AMPK pathway, up-regulate *p*-AMPK, stimulate the production of downstream PGC-1α proteins, and boost the functionality of the intracellular antioxidant enzymes SOD and GSH-Px, all the while diminishing the levels of MDA. Consequently, HFOs-AK regulates energy metabolism and delivers anti-fatigue benefits by stimulating the AMPK/PGC-1α pathway.

The Nrf2 protein plays a crucial role in regulating redox reactions within cells and controlling the expression of related factors downstream [59]. It is typically found in the cytoplasm, where it is bound to kelch-like ECH-associated protein 1 (Keap1) [60]. When ROS build up in the cell, Nrf2 separates from Keap1 and moves to the nucleus to bond with the antioxidant response element (ARE), which is overseen by the promoters of specific enzyme genes like heme oxygenase-1 (HO-1). Overall, the level of Nrf2 in cells is often inadequate to completely counteract the oxidative stress caused by physical exertion. Hence, the Keap1/Nrf2/ARE signaling pathway serves as a crucial target in combating fatigue and diseases related to oxidative stress. This pathway primarily operates by stimulating the production of phase II enzymes responsible for defending against oxidative stress and clearing ROS [45]. The results depicted in Figure 8 and Figure 9 clearly demonstrate a significant increase in Nrf2 protein expression within liver tissues of fatigued mice treated with HFOs-AK, indicating the activation of the Nrf2 pathway. This suggests that HFOs-AK could potentially alleviate fatigue through Nrf2 pathway activation.

Some natural products show good anti-fatigue function by modulating mitochondrial biological functions. For example, flavonoids extracted from parsley (*Petroselinum crispum*) can repair mitochondrial dysfunction by regulating PGC-1α and have good anti-fatigue activity [61]. Astragalus polysaccharide has the ability to repair mitochondrial malfunction by addressing issues with the fusion and division processes, while also decreasing the presence of PGC-1α [62]. Various research has shown that herbal remedies containing antioxidants possess effective anti-fatigue properties. Administering luteolin-6-C-Neohesperidoside helped restore depleted levels of HO-1 and Nrf2 due to exhaustive swimming [60]. Therefore, from Figure 9, it can be shown that in this experiment, the oxidative stress in vivo caused by the exhaustive swimming experiment in mice can change the morphology and function of mitochondria. In the Western blot analysis, HFOs-AK demonstrates its ability to maintain ATP levels in vivo by triggering the AMPK/PGC-1α/Nrf2 pathways (Figure 8). Furthermore, it enhances the functioning of antioxidant enzymes (SOD and GSH-Px) and reduces the presence of lipid peroxidation MDA, highlighting its promising anti-fatigue properties.

## 4. Materials and Methods

### 4.1. Materials and Chemical Reagents

HFOs-AK were prepared in our laboratory according to the previous method [5]. Whey peptides were purchased from Shanxi Feimi Biotechnology Co., Ltd. (Taiyuan, China). Assay kits for determination of lactylic acid (LA), hydrouric nitrogen (BUN), lactic acid dehydrogenase (LDH), creatine enzyme (CK), superoxide dioxidase (SOD), glutathione peroxide (GSH-Px), malondialdehyde (MDA), liver/plasmodium, and ATP were purchased from Nanjing Jiancheng Bioengineering Institute (Nanjing, China). The activity assay kits of Na^+^-K^+^-ATPase, Ca^2+^-Na^+^-K^+^-ATPase, and Ca^2+^-ATPase were purchased from Hangzhou Leyi Bio-technology Co., Ltd. (Hangzhou, China). AMPK alpha 2 Polyclonal antibody (18167-1-AP), PGC-1α monoclonal antibody (66369-1-Ig), GAPDHH antibody (MP50049-1), mouse antibody (HRP-10283), and rabbit anti-rat (IgG) antibody (ab6703) were bought from Sanying Bio-Biological Antibody Co., Ltd. (Wuhan, China).

### 4.2. Experimental Methods

#### 4.2.1. Animal Breeding and Experimental Design

Ninety male ICR mice (18–22 g) were purchased from Hangzhou Ziyuan Laboratory Animal Technology Co., Ltd. (Hangzhou, China). The animal experiments were standardized and approved by the Laboratory Animal Ethics Committee of Zhejiang Ocean University (certificate no. SYXK (Zhejiang) 2019–0031). The mice were kept at 25 ± 2 °C with 50 ± 5% relative humidity, following a 12-h light-dark cycle, and the mice had free access to food and water. After a week of acclimatization, male ICR mice were sorted into two groups: 10 mice in each of the 12 cages in group A and 5 mice in each of the 6 cages in group B. Blank group: mice were not gavaged. Model group: mice were injected with 0.2 mL distilled water for 30 days. Positive control group: male ICR mice received 0.2 mL whey peptides at a dose of 0.5 mg/g·bw/d for 30 days. HFOs-AK low-dose group: male ICR mice were treated with 0.2 mL HFOs-AK at a dose of 0.1 mg/g·bw/d for 30 days. HFOs-AK medium-dose group: Male ICR mice received 0.2 mL HFOs-AK at a dose of 0.3 mg/g·bw/d for 30 days. HFOs-AK high-dose group: male ICR mice received 0.2 mL HFOs-AK at a dose of 0.5 mg/g·bw/d for 30 days.

#### 4.2.2. Establishment of a Fatigue Model of Endurance Swimming Mice

According to the described method [23,63], 30 min after the final gavage in group A, the mice were introduced into a controlled swimming environment, where the water was maintained at a stable temperature of 25 °C and a depth of 30 cm. They were tasked with navigating a submerged wire structure while carrying a weight equivalent to 5% of their body weight. The water in the pool was continuously circulated throughout the swimming session. Duration of swimming was measured from the onset of activity until the mice were unable to resurface within 8 s after their heads submerged. This duration was documented as their swimming time.

#### 4.2.3. Measurement of Body Weight and Organ Index of Mice

After the beginning of the experiment, it was necessary to weigh and record the body weight of the mice at regular intervals every day, and all the mice were given mouse food daily according to the standard diet and free water. After the experiment, blood was removed from the mice’s eyeballs, and the liver, leg muscles, kidney, spleen, and thymus of the mice were taken for dissection. Each group of mice was weighed accurately on an analytical balance to calculate their organ index. The formula for calculating the organ index was as follows (1):

Organ index (%) = Weight of organs (g)/The mass of a mouse (g)(1)

#### 4.2.4. Histopathological Changes Observed

In male ICR mice, liver and muscle tissues were fixed overnight in 4% paraformaldehyde. Afterwards, paraffin is applied. Subsequently, H&E staining was performed on 4 mm sections of paraffin. A light microscope was used to observe the changes in histopathology.

#### 4.2.5. Measurement of Biochemical Indexes among Mice

Upon completion of 30 days of intervention, group B mice with ICR were subjected to blood sampling from the eyeballs immediately 30 min after the end of swimming, and the supernatant was centrifuged at 4000 rpm for 10 min and stored for further use. After the serum was taken, the mice were executed, and the liver and muscle of the mice were accurately weighed, added to a 0.9% NaCl solution, ground in an ice bath under a tissue homogenizer to prepare a 10% homogenate of the liver and muscle tissues of the mice, and centrifuged at 4000 rpm for 20 min. In addition to the supernatant of the tissues, oxidative stress indicators and changes of LA, BUN, BA, BG, MG, LG, LDH, CK, Na^+^-K^+^-ATP, CA^2+^-Mg^2+^-ATP, SOD, and GSH-Px were measured by a kit.

#### 4.2.6. Detecting the Expression of Relevant Signaling Pathway Proteins

A portion of liver tissue stored at −80 °C was used for Western blot analysis according to a previous method [64]. An RIPA lysis solution was used to homogenize and lyse liver tissue proteins, centrifuged at low temperature for 10 min at 12,000 rpm, and supernatants were taken, partitioned, and stored at −80 °C in a refrigerator for spare use. The total protein content in each group of samples was calculated by BCA protein assay according to the established method [63], and the calculated adjusted protein uploading volume was 5 μg/μL. Extracted proteins were uploaded onto a 12% SDS-acrylamide gel and then transferred onto a PVDF membrane. After incubation with primary and secondary antibodies, protein bands were visualized with an ECL luminescent solution. The quantitative analysis of the bands was performed by the Fluochem-FC3 system (Portsmouth, NH, USA) and statistically analyzed using SPSS (version 27.0).

### 4.3. Data Analysis

All data were expressed as means ± standard deviation (SD) (n = 15) and statistically analyzed using SPSS software for one-way ANOVA, LSD, and Duncan post hoc comparisons, and differences were considered statistically significant at *p* < 0.05.

## 5. Conclusions

In the study, we built an endurance swimming mice model to verify how HFOs-AK fights fatigue. The findings indicated that the HFOs-AK exhibited varying degrees of increased endurance during force exhaustion swimming, primarily attributable to two key factors. Regulating the AMPK signaling pathway induced a decrease in the levels of the metabolites LA, BUN, and BA and an increase in the contents of BG, MG, and LG in mice. Simultaneously, the decrease in LDH and CK leakage promoted the functions of Na^+^-K^+^-ATPase and Ca^2+^-Mg^2+^-ATPase, shielding the tissues from harm and boosting ATP levels within them. Regulatory of the Keap1/Nrf2/ARE signaling pathway led to a notable upsurge in SOD and GSH-Px functions and a drop in MDA concentrations in the hepatic tissue of mice, indicating the potential antioxidant properties of HFOs-AK. Therefore, HFOs-AK presented remarkable anti-fatigue function and can be used as a functional peptide applied in anti-fatigue food.

In addition, some relevant indicators, such as hemoglobin level, fecal SCFA content (propionic acid and butyric acid content), and intestinal flora changes, have not been comprehensively detected after force exhaustion swimming in mice, and the mechanism of HFOs-AK on attenuating muscle damage needs further study. More importantly, the efficacy and potential side effects of long-term use of HFOs-AK need to study in our follow-up experiments.

## Figures and Tables

**Table 1:** Changes in the body weight of the mice in each group (n = 15).

Groups	Body Weight (g)		
	Starting Weight	Final Weight	Weight Change
Blank	22.85 ± 1.00	37.81 ± 2.45	13.33 ± 1.73 ^b^
Model	22.96 ± 0.82	37.97 ± 3.17	15.01 ± 2.35 ^a,b^
Whey peptides	23.31 ± 0.72	37.71 ± 1.86	14.40 ± 1.14 ^a,b^
Low-dose HFOs-AK	22.91 ± 0.97	37.92 ± 1.22	15.01 ± 0.25 ^a,b^
Mid-dose HFOs-AK	21.53 ± 0.94	37.57 ± 0.66	16.03 ± 0.28 ^a,b^
High-dose HFOs-AK	22.38 ± 0.99	38.67 ± 2.60	16.29 ± 1.61 ^a^

All data are presented as the mean ± SD (n = 15). ^a,b^ Values with the same letters indicate no significant difference in each column (*p* > 0.05).

**Table 2:** Organ indices of mice in each group (n = 15).

Groups	Liver (%)	Spleen (%)	Gallbladder (%)	Thymus Gland (%)
Blank	5.95 ± 0.41 ^a^	0.32 ± 0.05 ^a^	1.59 ± 0.14 ^a^	0.15 ± 0.01 ^a^
Model	5.85 ± 0.12 ^a,b^	0.32 ± 0.01 ^a^	1.56 ± 0.04 ^a^	0.14 ± 0.01 ^a^
Whey peptides	5.59 ± 0.05 ^a,b^	0.31 ± 0.02 ^a^	1.55 ± 0.04 ^a^	0.13 ± 0.04 ^a^
Low-dose HFOs-AK	5.88 ± 0.13 ^a^	0.29 ± 0.02 ^a^	1.56 ± 0.09 ^a^	0.15 ± 0.06 ^a^
Mid-dose HFOs-AK	5.59 ± 0.23 ^b^	0.29 ± 0.01 ^a^	1.51 ± 0.05 ^a^	0.14 ± 0.04 ^a^
High-dose HFOs-AK	5.69 ± 0.03 ^a,b^	0.30 ± 0.03 ^a^	1.55 ± 0.03 ^a^	0.14 ± 0.05 ^a^

All data are presented as the mean ± SD (n = 15). ^a,b^ Values with the same letters indicate no significant difference in each column (*p* > 0.05).

## Data Availability

Data are contained within the article.

## Abbreviations

AAA: aromatic amino acids; ALT, alanine aminotransferase; AMPK, adenosine monophosphate activated protein kinase; ARE, antioxidant response element; AST, aspartate aminotransferase; ANOVA, analysis of variance; AMPK, Adenosine 5′-monophosphate (AMP)-activated protein kinase; BA, blood ammonia; BCAA, branched chain amino acid; BCA, bicinchoninic acid; BG, blood glucose; BUN, blood urea nitrogen; CK, creatine kinase; Da, dalton; ELISA, enzyme-linked immunosorbent assay; ECL, enhanced chemiluminescence; GSH-Px, glutathione peroxidase; HDL-C, high density lipoprotein cholesterol; H&E, hematoxylin-eosin staining; HFOs-AK, high Fischer ratio oligopeptides from Antarctic krill; HO-1, heme oxygenase-1; IκBα, Inhibitor Kappa Bα; LA, lactic acid; LDH, lactate dehydrogenase; LDL-C, low-density lipoprotein cholesterol; LG, liver glycogen; MDA, Malondialdehyde; MG, muscle glycogen; MW, molecular weight; NQO1, NAD(P)H: quinine oxidoreductase 1; Nrf2, nuclear factor erythroid 2-related factor 2; PPARα, peroxisome proliferator-activated receptor-α; PVDF, polyvinylidene difluoride; PGC-α, Peroxisome proliferator-activated receptor-γ coactivator; ROS, reactive oxygen species; SOD, superoxide dismutase; SREBP-1C, sterol regulatory element binding proteins-1c; SDS, Sodium dodecyl sulfate; SD, stable disease; SPSS, Statistical Package for the Social Sciences; SDS-PAGE sodium dodecyl sulfate polyacrylamide gel electrophoresis; TC, total cholesterol; TG, Triglyceride; TNF-α, tumour necrosis factor-α; TBS, Tris buffered saline; TBST, Tris buffered saline with Tween 20.

## References

[1] Abachi S., Offret C., Fliss I., Marette A., Bazinet L., Beaulieu L. (2022). Isolation of immunomodulatory biopeptides from Atlantic Mackerel (*Scomber scombrus*) protein hydrolysate based on molecular weight, charge, and hydrophobicity. Food Bioprocess Technol..

[2] Hu Y.D., Xi Q.H., Kong J., Zhao Y.Q., Chi C.F., Wang B. (2023). Angiotensin-I-converting enzyme (ACE)-inhibitory peptides from the collagens of monkfish (*Lophius litulon*) swim bladders: Isolation, characterization, molecular docking analysis and activity evaluation. Mar. Drugs.

[3] Quintal-Bojórquez N., Segura-Campos M.R. (2021). Bioactive peptides as therapeutic adjuvants for cancer. Nutr. Cancer.

[4] Wang Y.M., Zhang Z., Sheng Y., Chi C.F., Wang B. (2024). A systematic review on marine umami peptides: Biological sources, preparation methods, structure-umami relationship, mechanism of action and biological activities. Food Biosci..

[5] Lan C., Zhao Y.Q., Li X.R., Wang B. (2019). High Fischer ratio oligopeptides determination from Antartic krill: Preparation, peptides profiles, and in vitro antioxidant activity. J. Food Biochem..

[6] Tanabe S., Tanimoto S.Y., Watanabe M., Arai S. (1991). Nutritional effects of an oligopeptide mixture with a very high Fischer ratio on the amino acid absorption and cerebral amine metabolism in rats suffering from galactosamine-induced liver injury. Agric. Biol. Chem..

[7] Wang Z.G., Ying X.G., Wang Y.F., Yu X.W., Luo H.Y. (2019). Structural analysis and activity evaluation of high Fischer ratio oligopeptides from minced meat of skipjack (*Katsuwonus pelamis*). J. Aquat. Food Prod. Technol..

[8] Wang L., Sun J., Ding S., Qi B. (2018). Isolation and identification of novel antioxidant and antimicrobial oligopeptides from enzymatically hydrolyzed anchovy fish meal. Process Biochem..

[9] Niu R., Feng W. (2018). Research progress of phenylketonuria and its releveant treatment. Chin. New Drugs J..

[10] Liu R., Li Z., Yu X.-C., Hu J.-N., Zhu N., Liu X.-R., Hao Y.-T., Kang J.-W., Li Y. (2023). The effects of peanut oligopeptides on exercise-induced fatigue in mice and its underlying mechanism. Nutrients.

[11] Li D., Ren J.-W., Xu T., Li L., Liu P., Li Y. (2021). Effect of bovine bone collagen oligopeptides on wound healing in mice. Aging.

[12] Zheng S.L., Wang Y.Z., Zhao Y.Q., Chi C.F., Zhu W.Y., Wang B. (2023). High Fischer ratio oligopeptides from hard-shelled mussel: Preparation and hepatoprotective effect against acetaminophen-induced liver injury in mice. Food Biosci..

[13] Zhang X., Li S., Li M., Hemar Y. (2023). Study of the in vitro properties of oligopeptides from whey protein isolate with high Fisher’s ratio and their ability to prevent allergic response to β-lactoglobulin in vivo. Food Chem..

[14] Wang Y., Song X., Feng Y., Cui Q. (2019). Changes in peptidomes and Fischer ratios of corn-derived oligopeptides depending on enzyme hydrolysis approaches. Food Chem..

[15] Ying Z., Yuyang H., Meiying L., Bingyu S., Linlin L., Mingshou L., Min Q., Huanan G., Xiuqing Z. (2023). High Fischer ratio peptide of hemp seed: Preparation and anti-fatigue evaluation in vivo and in vitro. Food Res. Int..

[16] Salma N.U., Govindaraju K., Kumar B.S.G., Muthukumar S.P., Lakshmi A.J. (2022). Ameliorative effect of enhanced Fischer ratio flaxseed protein hydrolysate in combination with antioxidant micronutrients on ethanol-induced hepatic damage in a rat model. Br. J. Nutr..

[17] Zhao P., Hou Y., Chen X., Zhang M., Hu Z., Chen L., Huang J. (2024). High Fischer ratio oligopeptides of gluten alleviate alcohol-induced liver damage by regulating lipid metabolism and oxidative stress in rats. Foods.

[18] Qin Y., Cheng M., Fan X., Shao X., Wang C., Jiang H., Zhang X. (2022). Preparation and antioxidant activities of high Fischer’s ratio oligopeptides from goat whey. Food Sci. Anim. Resour..

[19] Zheng H., Zhang C., Cao W., Liu S., Ji H. (2009). Preparation and characterisation of the pearl oyster (*Pinctada martensii*) meat protein hydrolysates with a high Fischer ratio. Int. J. Food Sci. Technol..

[20] Xiong K., Liu J., Wang X., Sun B., Zhang Y., Zhao Z., Pei P., Li X. (2021). Engineering a carboxypeptidase from *Aspergillus niger* M00988 by mutation to increase its ability in high Fischer ratio oligopeptide preparation. J. Biotechnol..

[21] Feng Z., Wei Y., Xu Y., Zhang R., Li M., Qin H., Gu R., Cai M. (2022). The anti-fatigue activity of corn peptides and their effect on gut bacteria. J. Sci. Food Agric..

[22] Wang P., Zeng H., Lin S., Zhang Z., Zhang Y., Hu J. (2020). Anti-fatigue activities of hairtail (*Trichiurus lepturus*) hydrolysate in an endurance swimming mice model. J. Funct. Foods.

[23] Ye J., Shen C.H., Huang Y.Y., Zhang X.Q., Xiao M.T. (2017). Anti-fatigue activity of sea cucumber peptides prepared from *Stichopus japonicus* in an endurance swimming rat model. J. Sci. Food Agric..

[24] Fang L., Zhang R.X., Wei Y., Ling K., Lu L., Wang J., Pan X.C., Cai M.Y. (2022). Anti-fatigue effects of fermented soybean protein peptides in mice. J. Sci. Food Agric..

[25] Ge M.-X., Chen R.-P., Zhang L., Wang Y.-M., Chi C.-F., Wang B. (2023). Novel Ca-chelating peptides from protein hydrolysate of Antarctic krill (*Euphausia superba*): Preparation, characterization, and calcium absorption efficiency in Caco-2 cell monolayer model. Mar. Drugs.

[26] Li Y., Tan L., Liu F., Li M., Zeng S., Gui Y., Zhao Y., Wang J.J. (2023). Effects of soluble Antarctic krill protein-curcumin complex combined with photodynamic inactivation on the storage quality of shrimp. Food Chem..

[27] Wang Y.Z., Zhao Y.Q., Wang Y.M., Zhao W.H., Wang P., Chi C.F., Wang B. (2021). Antioxidant peptides from Antarctic Krill (*Euphausia superba*) hydrolysate: Preparation, identification and cytoprotection on H_2_O_2_-induced oxidative stress. J. Funct. Foods.

[28] Wang M., Zhang L., Yue H., Cai W., Yin H., Tian Y., Dong P., Wang J. (2023). Peptides from Antarctic krill (*Euphausia superba*) ameliorate acute liver injury in mice induced by carbon tetrachloride via activating the Nrf2/HO-1 pathway. Food Funct..

[29] Yue H., Li Y., Cai W., Bai X., Dong P., Wang J. (2022). Antarctic krill peptide alleviates liver fibrosis via downregulating the secondary bile acid mediated NLRP3 signaling pathway. Food Funct..

[30] Zheng J., Gao Y., Ding J., Sun N., Lin S. (2022). Antarctic krill peptides improve scopolamine-induced memory impairment in mice. Food Biosci..

[31] Liu Y., Lin S., Hu S., Wang D., Yao H., Sun N. (2022). Co-administration of Antarctic krill peptide EEEFDATR and calcium shows superior osteogenetic activity. Food Biosci..

[32] Hatanaka A., Miyahara H., Suzuki K.I., Sato S. (2009). Isolation and identification of antihypertensive peptides from Antarctic krill tail meat hydrolysate. J. Food Sci..

[33] Li X., Zhang H., Xu H. (2009). Analysis of chemical components of shiitake polysaccharides and its anti-fatigue effect under vibration. Int. J. Biol. Macromol..

[34] Glancy B., Kane D.A., Kavazis A.N., Goodwin M.L., Willis W.T., Gladden L.B. (2021). Mitochondrial lactate metabolism: History and implications for exercise and disease. J. Physiol..

[35] Zhao R., Wu R., Jin J., Ning K., Wang Z., Yi X., Kapilevich L., Liu J. (2023). Signaling pathways regulated by natural active ingredients in the fight against exercise fatigue—A review. Front. Pharmacol..

[36] Liu R., Wu L., Du Q., Ren J.W., Chen Q.H., Li D., Mao R.X., Liu X.R., Li Y. (2018). Small molecule oligopeptides isolated from walnut (*Juglans regia* L.) and their anti-fatigue effects in mice. Molecules.

[37] Zhong L., Zhao L., Yang F., Yang W., Sun Y., Hu Q. (2017). Evaluation of anti-fatigue property of the extruded product of cereal grains mixed with *Cordyceps militaris* on mice. J. Int. Soc. Sports Nutr..

[38] Lu X., Chen J., Huang L., Ou Y., Wu J., Guo Z., Zheng B. (2023). The anti-fatigue effect of glycoprotein from Hairtail fish (*Trichiurus lepturus*) on BALB/c mice. Foods.

[39] Yu Y., Wu G., Jiang Y., Li B., Feng C., Ge Y., Le H., Jiang L., Liu H., Shi Y. (2020). Sea cucumber peptides improved the mitochondrial capacity of mice: A potential mechanism to enhance gluconeogenesis and fat catabolism during exercise for improved antifatigue property. Oxidative Med. Cell. Longev..

[40] Kozakowska M., Pietraszek-Gremplewicz K., Jozkowicz A., Dulak J. (2015). The role of oxidative stress in skeletal muscle injury and regeneration: Focus on antioxidant enzymes. J. Muscle Res. Cell Motil..

[41] Suo S.K., Zheng S.L., Chi C.F., Luo H.Y., Wang B. (2022). Novel angiotensin-converting enzyme inhibitory peptides from tuna byproducts-milts: Preparation, characterization, molecular docking study, and antioxidant function on H_2_O_2_-damaged human umbilical vein endothelial cells. Front. Nutr..

[42] Liu S., Wang M.Y., Xing Y.B., Wang X.R., Cui C.B. (2023). Anti-oxidation and anti-fatigue effects of the total flavonoids of *Sedum aizoon* L.. J. Agric. Food Res..

[43] Zou D., Liu P., Chen K., Xie Q., Liang X., Bai Q., Zhou Q., Liu K., Zhang T., Zhu J. (2015). Protective effects of myricetin on acute hypoxia-induced exercise intolerance and mitochondrial impairments in rats. PLoS ONE.

[44] Zou D., Chen K., Liu P., Chang H., Zhu J., Mi M. (2014). Dihydromyricetin improves physical performance under simulated high altitude. Med. Sci. Sports Exerc..

[45] Wang X., Qu Y., Zhang Y., Li S., Sun Y., Chen Z., Teng L., Wang D. (2018). Antifatigue potential activity of *Sarcodon imbricatus* in acute excise-treated and chronic fatigue syndrome in mice via regulation of Nrf2-mediated oxidative stress. Oxidative Med. Cell. Longev..

[46] Fu X., Ji R., Dam J. (2010). Antifatigue effect of coenzyme Q10 in mice. J. Med. Food.

[47] Osman W.N.W., Mohamed S. (2018). Standardized *Morinda citrifolia* L. and *Morinda elliptica* L. leaf extracts alleviated fatigue by improving glycogen storage and lipid/carbohydrate metabolism. Phytother. Res..

[48] Lee M.C., Hsu Y.J., Lin Y.Q., Chen L.N., Chen M.T., Huang C.C. (2022). Effects of perch essence supplementation on improving exercise performance and anti-fatigue in mice. Int. J. Environ. Res. Public Health.

[49] Falavigna G., Junior J.A.D.A., Rogero M.M., Pires I.S.D.O., Pedrosa R.G., Junior E.M., de Castro I.A., Tirapegui J. (2012). Effects of diets supplemented with branched-chain amino acids on the performance and fatigue mechanisms of rats submitted to prolonged physical exercise. Nutrients.

[50] Guo Z., Lin D., Guo J., Zhang Y., Zheng B. (2017). In vitro antioxidant activity and in vivo anti-fatigue effect of sea horse (*Hippocampus*) peptides. Molecules.

[51] Lv J.J., Liu Y., Zeng X.Y., Yu J., Li Y., Du X.Q., Wu Z.B., Hao S.L., Wang B.C. (2021). Anti-fatigue peptides from the enzymatic hydrolysates of *Cervus elaphus* blood. Molecules.

[52] Cai B., Yi X., Wang Z., Zhao X., Duan A., Chen H., Wan P., Chen D., Huang J., Pan J. (2023). Anti-fatigue effects and mechanism of *Syngnathus schlegeli* peptides supplementation on exercise-fatigued mice. J. Funct. Foods.

[53] Ren Y., Wu H., Chi Y., Deng R., He Q. (2020). Structural characterization, erythrocyte protection, and antifatigue effect of antioxidant collagen peptides from tilapia (*Oreochromis nilotica* L.) skin. Food Funct..

[54] Feng R., Zou X., Wang K., Liu H., Hong H., Luo Y., Tan Y. (2023). Antifatigue and microbiome reshaping effects of yak bone collagen peptides on Balb/c mice. Food Biosci..

[55] Wang X.Q., Yu H.H., Xing R.G., Liu S., Chen X.L., Li P.C. (2023). Structural properties, anti-fatigue and immunological effect of low molecular weight peptide from monkfish. J. Funct. Foods.

[56] Ke R., Xu Q., Li C., Luo L., Huang D. (2018). Mechanisms of AMPK in the maintenance of ATP balance during energy metabolism. Cell Biol. Int..

[57] Shrikanth C.B., Nandini C.D. (2020). AMPK in microvascular complications of diabetes and the beneficial effects of AMPK activators from plants. Phytomedicine.

[58] Zhang L., Zhou Y., Wu W., Hou L., Chen H., Zuo B.O., Xiong Y., Yang J. (2017). Skeletal muscle-specific overexpression of PGC-1α induces fiber-type conversion through enhanced mitochondrial respiration and fatty acid oxidation in mice and pigs. Int. J. Biol. Sci..

[59] Cai W.-W., Hu X.-M., Wang Y.-M., Chi C.-F., Wang B. (2022). Bioactive peptides from Skipjack tuna cardiac arterial bulbs: Preparation, identification, antioxidant activity, and stability against thermal, pH, and simulated gastrointestinal digestion treatments. Mar. Drugs.

[60] Duan F.F., Guo Y., Li J.W., Yuan K. (2017). Antifatigue effect of luteolin-6-C-neohesperidoside on oxidative stress injury induced by forced swimming of rats through modulation of Nrf2/ARE signaling pathways. Oxidative Med. Cell. Longev..

[61] Wang Y., Zhang Y., Hou M., Han W. (2022). Anti-fatigue activity of parsley (*Petroselinum crispum*) flavonoids via regulation of oxidative stress and gut microbiota in mice. J. Funct. Foods.

[62] Huang Y.F., Lu L., Zhu D.J., Wang M., Yin Y., Chen D.X., Wei L.B. (2016). Effects of astragalus polysaccharides on dysfunction of mitochondrial dynamics induced by oxidative stress. Oxidative Med. Cell. Longev..

[63] Zhao C., Gong Y., Zheng L., Zhao M. (2023). Whey protein hydrolysate enhances exercise endurance, regulates energy metabolism, and attenuates muscle damage in exercise mice. Food Biosci..

[64] Zheng S.L., Wang Y.M., Chi C.F., Wang B. (2024). Chemical characterization of honeysuckle polyphenols and their alleviating function on ultraviolet B-damaged HaCaT cells by modulating the Nrf2/NF-κB signaling pathways. Antioxidants.

